# A survey on brachytherapy training of gynecological cancer focusing on the competence of residents in China

**DOI:** 10.1186/s13014-024-02433-6

**Published:** 2024-05-21

**Authors:** Mohan Dong, Changhao Liu, Junfang Yan, Yong Zhu, Yutian Yin, Jia Wang, Ying Zhang, Lichun Wei, Lina Zhao

**Affiliations:** 1 Department of Medical Education, Xijing Hospital, Air Force Medical University, Xi‘an, China; 2 Department of Radiation Oncology, Xijing Hospital, Air Force Medical University, Xi‘an, China; 3 Department of Radiation Oncology, Peking Union Medical College Hospital, Chinese Academy of Medical Science and Peking Union Medical College, Beijing, China; 4 Department of Radiation Oncology, Baoji Central Hospital, Shaanxi, China; 5 Department of Radiation Oncology, the Affiliated Hospital of Yan’an University, Yanàn, China

**Keywords:** Brachytherapy training, Standardized training for residents, The competency-based medical education (CBME), Gynecological cancer

## Abstract

**Background:**

The brachytherapy is an indispensable treatment for gynecological tumors, but the quality and efficiency of brachytherapy training for residents is still unclear.

**Methods:**

An anonymous questionnaire was designed to collect information on gynecological brachytherapy (GBT) training for radiation oncology residents from 28 training bases in China. The questionnaire content was designed based on the principle of competency based medical education (CBME). The Likert scale was employed to evaluate self-reported competence and comprehension regarding GBT. A total of 132 senior residents were included in the final analysis.

**Results:**

53.79% (71/132) of senior residents had experience in performing image-guided GBT, whereas 76.52% (101/132) had observed the procedure during their standardized residency training. The proportion of senior residents who reported having the self-reported competence to independently complete the GBT was 78.03% for intracavity GBT, 75.00% for vaginal stump GBT, and 50.03% for interstitial GBT, respectively. The number of successful completion of Interstitial, intracavity and vaginal GBT was correlated with the self- confidence of trainees after standardized training. In particular, the independent completion of interstitial GBT for more than 20 cases was an independent factor for the self-reported competence of senior residents. During the training period, 50.76% and 56.82% of the residents had not participated in the specialized examinations and professional GBT courses.

**Conclusions:**

The study revealed that the self-confidence of residents to independently complete brachytherapy was relatively high, and the specialized curriculum setting and training process assessment for brachytherapy training still need to be strengthened in the future.

**Supplementary Information:**

The online version contains supplementary material available at 10.1186/s13014-024-02433-6.

## Background

Brachytherapy is an essential mode of treatment for gynecological tumors, specifically cervical cancer and endometrial cancer [1, 2]. Compared to patients with locally advanced cervical or endometrial cancer who did not undergo GBT, there was a significant survival benefit in those patients treated with GBT. In particular, image-guided adaptive brachytherapy (IGBT) combined with interstitial techniques has been demonstrated to enhance local control and survival for patients with cervix cancer, while reducing the incidence of complications. However, several studies indicated that the utilization rate of GBT was declining or disparities [3]. Strengthening the training and education of resident doctors on brachytherapy is one of the important methods to improve the application of GBT.

The competency-based medical education (CBME) has emerged as a crucial training model during residency training [4–6]. The clinical competence of residents encompasses patient care, medical knowledge, professionalism, system-based practice, practice-based learning, and communication skill. The assessment of competence would still be based on entrustable professional activity (EPA) worldwide. In China, the residents should undergo a standardized residency training program of three years’ duration, after which they could be appointed as staff to conduct professional training for a period of over two years [7, 8]. The training provided to residents emphasizes on enhancing their skills and knowledge, as well as improving their accountability and proficiency.

According to a survey regarding GBT training in Europe and the United States, only 35% of students in Europe and 59% of students in the United States gained the confidence they needed to independently operate GBT upon completion of the training. Additionally, only 35% of students in both regions passed the corresponding institutional examinations [9, 10]. In China, all institutes for radiotherapy training are equipped with GBT treatment machines and treat over 100 patients with gynecological tumors each year [7, 11]. The residents who specialize in radiation therapy are mandated to manage more than 10 patients with gynecological tumors requiring radiotherapy. However, the current status of GBT training in gynecologic cancer for radiation oncology residents and the factors related to their competence in completing GBT were not clear.

GBT training has its own characteristics. During GBT training, the residents were required to understand the distinctive physical characteristics and biological function of brachytherapy, the principles of target delineation and dose evaluation system, and also to practice placing applicators in a reasonable manner. This study has investigated issues related to GBT training for residents specializing in radiation therapy with aim to find ways for improving the process and outcome of training.

## Methods

In order to assess the current status of GBT training in China, an anonymous questionnaire was designed and sent to 28 institutes nationwide on the beginning of December 2022. The response for questionnaire sheets including personnel information and 20 questions related to GBT training were completed within a week and submitted to the department of radiation oncology of the Xijing hospital via email. The questionnaire (list in appendix 1) included the self-reported assessment of competence in performing GBT, institutional support, barriers to acquiring competence, and preferences for additional training. The survey encompassed the competence on three ways of vaginal stump brachytherapy, intracavity brachytherapy, and interstitial brachytherapy, also included the practice on image-guided GBT.

A semi-quantitative evaluation was used for certain content to reflect the strength of opinion, by using a 5-Likert-type scale, which included labels such as “very irrelevant,” “unimportant,” and “impossible,” as well as “very relevant,” “important,” and “possible.” The self-reported competence was classified as follows: unable to perform GBT, competence to perform GBT with major assistance, indeterminacy, competence to perform GBT with minor assistance, and competence to perform GBT whole independently. During statistical analysis, we classify the ability to perform GBT with minor assistance or complete it entirely independently as having strong confidence to complete GBT. The scale for practice or experience was determined by analyzing the workload of units investigated and reports of previous papers [9, 10]. The papers read by residents should include the ICRU 89 report, the MRI-guided brachytherapy guidelines, the ABC guidelines, and the Chinese expert consensus [12– 14].

The Statistical Package for Social Sciences (SPSS version 27.0, IBM, Armonk, NY, USA) software was employed for the statistical analyses. Continuous variables, categorical variables and inter-group differences were processed according to statistical principles. The univariable and multivariable logistic regression analysis was used to investigate the influence factors on self-reported competence. The factors that reached statistical significance after screening by univariable logistic regression analysis (with a *p* value of 0.05 for entry and 0.10 for removal) were included in the multivariable logistic regression analysis model, and estimated hazard ratios (HRs) and 95% confidence intervals (CIs) were calculated.

## Results

### Participants and response to questionnaire

One hundred and ninety-nine residents special for radiation oncology from 28 hospitals had completed anonymous questionnaires. Of the participants, 100 trainees were in their standardized residency training year, while 99 others were in professional training for radiotherapy. To ensure a more comprehensive perspective and representativeness of the results from the present study, the statistical analysis was restricted to 132 participants, namely senior residents, including 33 residents in their third year of standardized training and 99 others in professional training.

**Fig. 1:**
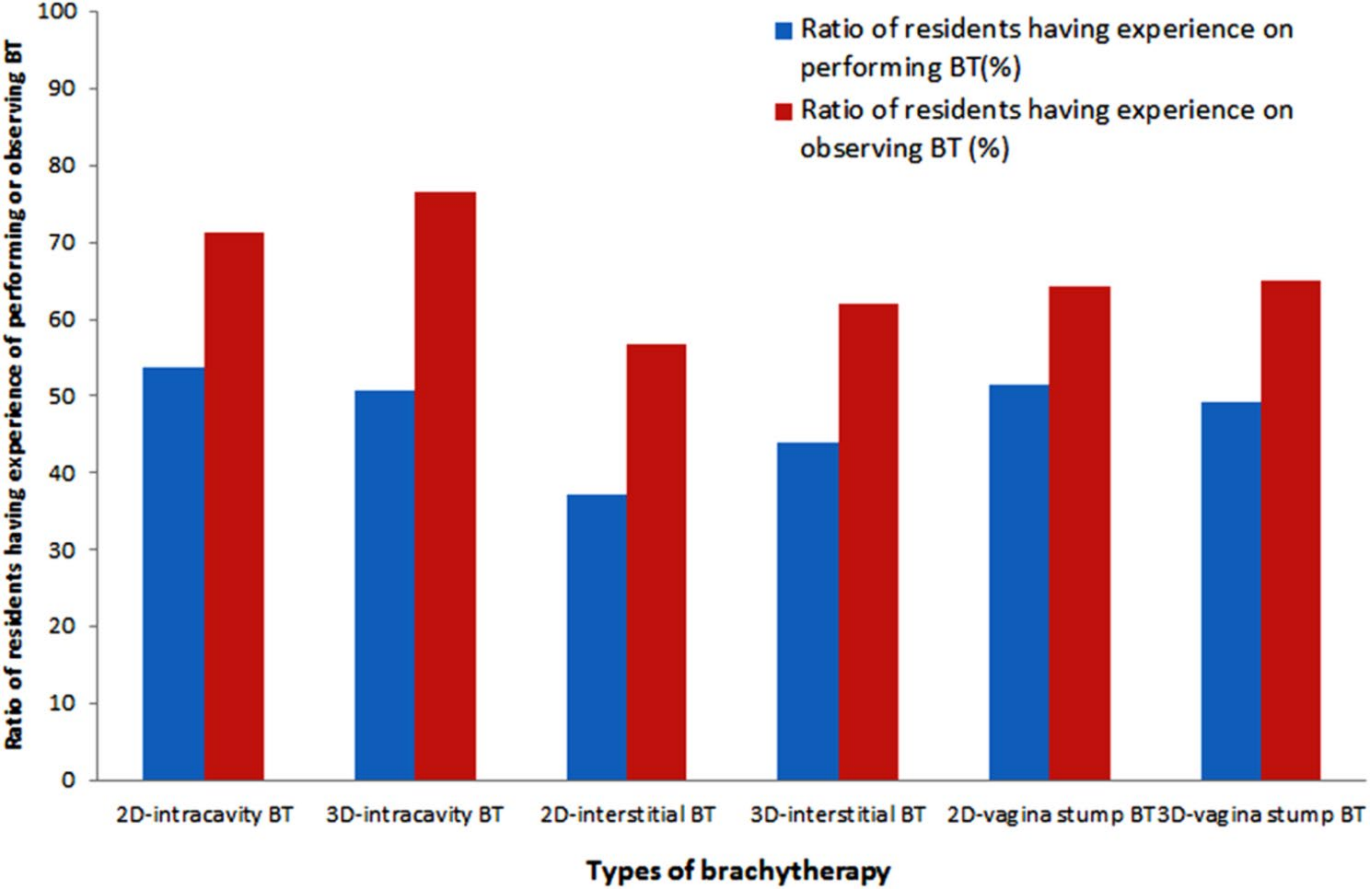
The percentage of senior residents having practice or observation on GBT process

The number of gynecological cancer cases across all investigated training units exceeds 100 cases per year, with a median of 600 cases (range 180–1200) per year. According to the data presented in Fig. 1, 53.79% (71/132) of senior residents had experience in performing image-guided GBT, and 76.52% (101/132) had observed the process. 91.67% (121 out of 132) of the residents took care of 10 or more patients with gynecological tumors. 92.42% (122 out of 132) of the residents read MRI images of 10 or more patients with gynecological tumors. 97.73% (129/132) of residents have read professional articles about GBT. 56.82% (75/132) of the residents have participated in the formal courses of GBT. During training period, 93.18% (123 out of 132) of them participated in more than one international or domestic academic activity. Table 1 contains detailed information on the resident’s understanding of GBT and related issues in this survey.

**Table 1:** The response from senior residents to issues investigated

Variables (questions)	% (n of N)
Residents caring 10 or more patients with gynecological tumor	91. 67% (121/132)
Residents reading MRI image for 10 or more patients with gynecological tumor	92.42% (122/132)
Residents reading professional article about GBT	97.73%(129/132)
Residents participating in more than one international or domestic academic activities	93. 18% (123/132)
Residents participating in a GBT curriculum special for training during their residency program	56. 82%(75/132)
Residents attending special evaluation for GBT training	46.97%(62/132)
Residents with high or somewhat high confidence to start a SBRT practice	33.33% (44/132)
Residents realizing that performing 2D/3D GBT* independently at the end of residency was “very or somewhat” important	96.97%(128/132)
Residents realizing that the leader of the residential training program played an important role in GBT training	95.46% (126/132)
Residents believing that the application of brachytherapy in cervical cancer would increase or remain unchanged in the future	91.67% (121/132)
Residents believing that the application of brachytherapy in endometrial cancer would increase or remain unchanged in the future	89.39% (118/132)
Residents considering that greatest barrier to achieving GBT independence at the end of residency training was lack of training	33.33%(44/132)
Residents considering that greatest barrier to achieving GBT independence at the end of residency was lack of interest	12.12% (16/132)
Residents considering that simulation phantom should be used for training	93.94% (124/132)
Residents considering that special brachytherapy courses for training should be set up	91.67% (121/132)

*3D GBT, image guided GBT

### The self-reported competence about finishing brachytherapy of gynecologic cancer

The percentage of senior residents who were confident to complete the GBT independently was 78.03% (103/132) for intracavitary and 75.00% (99/132) for vaginal stump brachytherapy respectively. However, for interstitial implantation, the percentage was only 50.03% (70/132).

The factors related to self-reported competence of senior residents in finishing brachytherapy were listed in Tables 2 and 3 and supplementary Tables 1–4, including GBT load of units, the number of reading literature, patient management, image analysis and the number for observation and practice of GBT. The univariate and multivariate analysis revealed a significant association between the competence in performing interstitial GBT independently and the number of cases operated, as well as the GBT load of institutes. In the multivariate analysis of confidence in completing vaginal and intracavitary brachytherapy, only the number of observing GBT was significantly correlated with confidence (as shown in supplementary Tables 1–4).

**Fig. 2:**
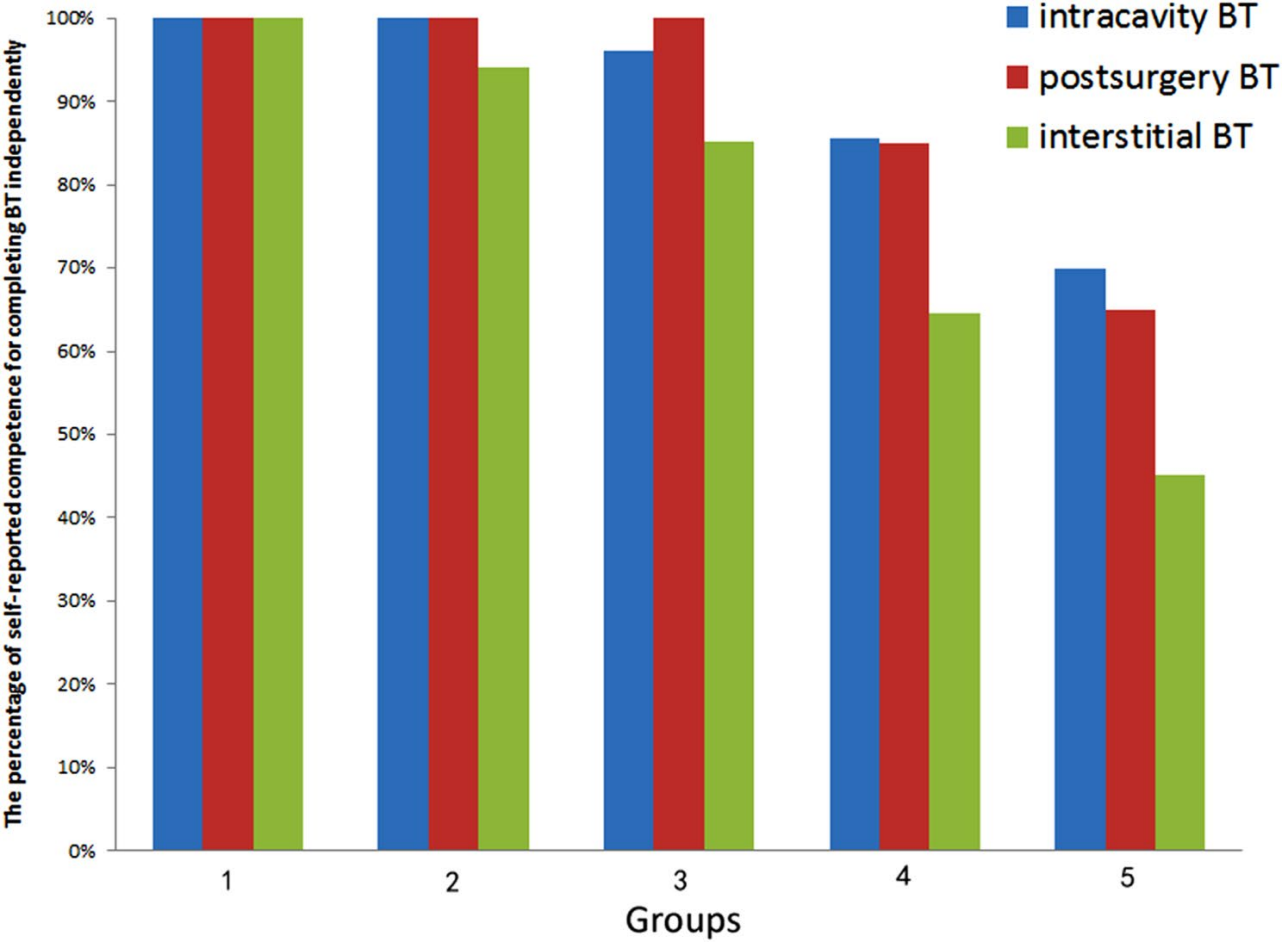
The relationship between the self-reported competence in completing BT and the number of BT practices performed. Groups 1–5 represent 20 cases, 10 cases, 5 cases, 1 case and 0 cases of BT performed by residents

The residents with more operational experience exhibited greater competence in executing GBT. According to the self-report, all senior residents are comfident in performing GBT as long as they have completed 10 cases of intracavitary, 5 cases of vaginal stump, or 20 cases of interstitial implantation (as shown in Fig. 2 and supplementary Table 5).

**Table 2:** The factors related with competence of senior residents in finishing interstitial brachytherapy (IBT) independently [M (range)/ n (%)]

Variables	Group with strong confidence^#^(*N* = 70)	Group without confidence(*N* = 62)	p
Workload of brachytherapy in training unit(cases/year)	900(180–1200)	360(180–1200)	0.009
IBT cases performed by residents			0.005
≤ 20	59(84.3)	61(98.4)	
> 20	11(15.7)	1(1.6)	
IBT cases observed by residents			0.001
≤ 20	53(75.7)	60(96.8)	
> 20	17(24.3)	2(3.2)	
Cases cared by residents			0.366
≤ 20	24(34.3)	26(41.9)	
> 20	46(65.7)	36(58.1)	
MRI images analyzed by residents			0.464
≤ 20	25(35.7)	26(41.9)	
> 20	45(64.3)	36(58.1)	
papers about brachytherapy read by residents			0.222
≤ 10	39(55.7)	41(66.1)	
> 10	31(44.3)	21(33.9)	
Residents attending brachytherapy curriculum			0.137
Yes	44(62.9)	31(50.0)	
No	26(37.1)	31(50.0)	
Residents with or without interesting for brachytherapy			0.498
Without	12(17.1)	8(12.9)	
With	58(82.9)	54(87.1)	
Residents attending academic activities (times)			0.903
≤2	32(45.7)	29(46.8)	
>2	38(54.3)	33(53.2)	
Residents taking brachytherapy very serious			0.890
No	2 (2.9)	3(4.8)	
Yes	68(97.1)	59(95.2)	

^#^: strong confidence to complete GBT was classified as the ability to perform GBT with minor assistance or complete it entirely independently

**Table 3:** Univariate and multivariate logistic regression analysis for competence of senior residents in finishing IBT independently

Variables	Univariate		Multivariate	
	HR (95%CI for HR)	p	HR (95%CI for HR)	p
Workload of brachytherapy in training unit(cases/year)	0.998(0.997–0.999)	0.003	0.999(0.998-1.000)	0.020
IBT cases performed by residents				
≤ 20	ref		ref	
> 20	11.373(1.423–90.867)	0.022	7.603(1.647–35.099)	0.009
IBT cases observed by residents				
≤ 20	ref			
> 20	9.623(2.124–43.604)	0.003		
Cases cared by residents				
≤ 20	ref			
> 20	1.384(0.683–2.804)	0.366		
MRI images analyzed by residents				
≤ 20	ref			
> 20	1.300(0.644–2.624)	0.464		
papers about brachytherapy read by residents				
≤ 10	ref			
> 10	1.552(0.766–3.145)	0.223		
Residents attending brachytherapy curriculum				
No	ref			
Yes	1.692(0.845–3.391)	0.138		
Residents with or without interesting for brachytherapy				
Without	ref			
With	0.716(0.272–1.886)	0.499		
Residents attending academic activities (times)				
≤ 2	ref			
>2	1.044(0.526–2.071)	0.903		
Residents taking brachytherapy very serious				
No	ref			
Yes	1.729(0.279–10.701)	0.556		

Abbreviations: Hazard ratios (HR) and 95% confidence intervals (CI) were calculated by a stratified logistic proportional hazards model

### The resident`s realization on the importance of GBT

The residents had the highest assurance in managing patients with gynecologic cancer compared to others (see supplementary Table 6). Compared to completing stereotactic body radiation therapy (SBRT) (44/132, 33.3%), a higher proportion of residents could independently complete GBT (50.03–78.03%). Out of the 132 residents surveyed, 60.61% (80/132) of the population expressed their opinion that the reduction in GBT usage was a substantial concern that impacted the treatment outcomes. In the treatment of cervical cancer and endometrial cancer, 91.67% and 89.39% of respondents respectively believed that the use of GBT would remain consistent, or even grow in the future.

### The existing problems and expectations for improving GBT training

21.97% (29/132) of residents believed that they did not have sufficient operational opportunities, despite the high GBT workload in the institutes. Regarding entrustability for finishing brachytherapy, 50.76% (67/132) of residents confirmed that no specific tests for GBT were conducted during their training.

To enhance the effectiveness of GBT training, the residents realized that it was crucial to establish a dedicated assessment process that was aligns with the content of training. 91.67%(121/132) residents believed that it was necessary to establish comprehensive GBT courses special for training. 93.94% (124/132) of residents believed that simulation phantoms should be used for training purposes.

## Discussion

It was the first investigation about gynecological tumor brachytherapy training for residents in China. The current study revealed that among senior residents, 78.03%, 75%, and 50.03% of participants had the self-reported competence to perform intracavity, vaginal stump and interstitial brachytherapy on their own. 46.99% of the residents passed the special GBT ability assessment. The results further suggested that for residents to gain confidence in GBT, a minimum of 10, 5, 20 cases respectively for intracavity, vaginal stump, interstitial GBT practice were required. To enhance the quality of GBT training, the special and comprehensive curriculum along with assessment for entrustability is necessary.

The incidence of gynecological tumors, including cervical cancer, remained high in China [15]. The GBT workload for gynecological tumors investigated in this study’s training institutes ranged from 180 to 1200 cases per year, offering ample opportunities for trainees to engage in operations and observe clinical procedures. Therefore, the self-confidence in completing the GBT of cervical or endometrial cancer is relatively high when compared to other studies [9, 10]. According to EMBRACE research, image-guided GBT can enhance the local control rate and survival [16]. GBT is considered an indispensable method for the treatment of cervical cancer in international treatment guidelines. According to Chinese expert consensus [17], patients in units without GBT procedures must be promptly referred to department capable of performing GBT. In this survey, 91.67% and 89.39% of residents believed that the application of GBT for cervical cancer and endometrial cancer would not decrease in the future. 96.48% of the residents strongly believed that the GBT training was extremely valuable for the standardized treatment of gynecological tumors.

A positive correlation was discovered between the number of cases in GBT practice and the self-reported competence of residents. The residents training for radiation oncology in China were mandated to care more than 40 patients who required radiotherapy, being more than 10 with gynecological tumors among them [7]. The Accreditation Council for Graduate Medical Education (ACGME) mandates that residents in radiation oncology must carry out a minimum of 5 interstitial and 15 intracavitary procedures throughout their residency training. The survey conducted in the United States reveals that individuals with 15 or more experiences of treatment have significantly high confidence in finishing brachytherapy [10]. European studies also revealed that while 50% of the residents believed that individuals who completed 15 cases of intracavitary brachytherapy could have high confidence, 87% of the residents acknowledged that those who practice 5 cases of interstitial implantation could not independently accomplish such complex brachytherapy [9]. The current study discovered that providing training on finishing more than 10 cases of intracavity, 5 cases of vaginal stump, and 20 cases of interstitial brachytherapy could aid residents in developing substantial self-reported competence.

The ideas behind competency-based training held immense significance for medical education. The competencies should be specific, comprehensive, and trainable [4]. The competence of brachytherapy was a crucial element of the ACGME milestones for radiation oncology medical residents [18, 19]. Performing GBT well requires not only knowledge and skill related, but also professional quality, empathetic patient care, communication, and cooperation. In China, there are textbooks specifically written for residents in the field of radiotherapy including the knowledge of GBT [20]. Our research also indicates that reading more papers and observing more GBT process can enhance confidence to finish interstitial operation, albeit without statistical difference.

The assessment of competence is a fundamental aspect of EPAs [21–23]. The purpose of formative assessment is to identify any problems that exist in students’ knowledge, skills, and attitudes. According to results of studies about medical training [24, 25], implementing high-quality formative evaluation according to a schedule and criterion can enhance the outcome of summative assessment and improve the competence of residents. The assessment of EPAs can also assist supervisors in determining the entrustability of their trainees. Despite this, our study found that 50.76% of individuals did not participate in the special assessment for GBT training. Establishing a standardized test system special for GBT training and supervising the procession in the future is an urgent problem.

There are numerous methods available for clinical skill training, including Chart-stimulated recall, direct observation, clinical vignettes, and multisource feedback. The method of simulator training is highly efficient [26, 27]. For instance, the seven-hour endoscopic simulation operation training has considerably enhanced the ability of the actual operation in the operating room [28]. In the European study, only 36% of the residents were found to have operated on more than 5 cases of cervical cancer’s brachytherapy. Our research indicated that despite the high workload of GBT in training bases, 21.97% of residents believed that they did not get sufficient GBT operational opportunities. Due to the limited rotation time in the radiotherapy department, it was impossible to utilize actual patients to enhance the confidence of all residents in operating. Hence, the development of a simulation phantom for training is highly imperative. Campelo and his team designed and produced a simulation phantom for the GBT training by utilizing 3D printing technology [29]. The phantom well exhibited human histological characteristics and was suitable for exercising intracavitary and interstitial brachytherapy, as well as for teaching and practicing image-guided brachytherapy. The phantom training is expected to enhance the resident` s competence and training efficiency.

There are certain limitations present in this study. Firstly, small sample size and selection bias should be found in this study. However, considering the actual number of residents and training bases (workload of 180–1200 cases per year) investigated, the result of the study was somewhat representative. Secondly, only self-evaluation indicators are employed to demonstrate competence in completing GBT in the study. In the future, we aim to create a detailed collection of assessment criteria and teaching quality control measures special for the theory and practice of brachytherapy training. Thirdly, the impact of trainer-related elements on the training outcome was not investigated and analyzed. Recently, there have been numerous trainings and seminars conducted for teachers, with a focus on residency training across the country. It is expected that the quality and efficiency of training will be enhanced in the future by implementing normative training system for both the trainer and the trainee.

## Conclusion

The study revealed that the self-reported competence in performing GBT was relatively high among the surveyed residents who specialized in radiation therapy. However, in the future, it is important to strengthen the scheduling of a comprehensive curriculum, assessment procedure special for GBT training, and provision of more practice opportunities and teaching devices.

### Electronic supplementary material

Below is the link to the electronic supplementary material.

Supplementary Material 1

## Data Availability

No datasets were generated or analysed during the current study.

## Abbreviations

GBT: Gynecological brachytherapy
IGBT: image-guided adaptive brachytherapy
CBME: competency-based medical education
IBT: interstitial brachytherapy
CI: 95% confidence intervals
HR: Hazard ratio
SBRT: stereotactic body radiation therapy
EMBRACE: IntErnational study on MRI-guided BRAchytherapy in CErvical cancer
ACGME: Accreditation Council for Graduate Medical Education
EPAs: entrustable professional activities
