# Supplementary material for: A survey on brachytherapy training of gynecological cancer focusing on the competence of residents in China
Source: Radiat Oncol. 2024 May 21;19:60. doi: 10.1186/s13014-024-02433-6 (PMC11110279; doi:10.1186/s13014-024-02433-6)
Supplement: Supplementary file 1 — Supplementary Material 1 [file 13014_2024_2433_MOESM1_ESM.docx]

**A survey on brachytherapy training of gynecological cancer focusing on the self-reported competence of residents specialized radiation oncology.**

Mohan Dong^#^, Ph.D; Changhao Liu^#^, MD; Junfang Yan, MD; Yong Zhu, MD; Yutian Yin, MD; Jia Wang, MD; Ying Zhang^a🖂^, MD; Lichun Wei^a🖂^, MD; Lina Zhao^a🖂^, MD

Tab. data in this file

| **contents** | **page** |
| --- | --- |
| **supplementary data** | **2** |
| **supplementary data: Appendix-1** | **11** |
| **supplementary data: Appendix-2** | **22** |

**Supplementary data**

Table 1. The factors related with self-reported competence of senior residents in finishing cavity brachytherapy independently [M (range)/ n (%)]

| Variables | Group with strong confidence^#^ (N=103) | Group without confidence(N=29) | *p* |
| --- | --- | --- | --- |
| Workload of brachytherapy in SR-training unit(cases/year) | 600(180-1200) | 720(180-1050) | 0.904 |
| Performing cavity brachytherapy cases (3D) |  |  | <0.001 |
| ≤10 | 77(74.8) | 29(100.0) |  |
| >10 | 26(25.2) | 0(0) |  |
| Observating cavity brachytherapy cases (3D) |  |  | 0.627 |
| ≤10 | 59(57.3) | 27(93.1) |  |
| >10 | 44(42.7) | 2(6.9) |  |
| Take caring of cases |  |  | 0.488 |
| ≤10 | 22(21.4) | 5(17.2) |  |
| >10 | 81(78.6) | 24(82.8) |  |
| Reading MRI image cases |  |  | 0.540 |
| ≤10 | 20(19.4) | 4(13.8) |  |
| >10 | 83(80.6) | 25(86.2) |  |
| Reading papers about brachytherapy |  |  | 0.284 |
| ≤10 | 61(59.2) | 19(65.5) |  |
| >10 | 42(40.8) | 10(34.5) |  |
| Attending brachytherapy curriculum |  |  | 0.358 |
| Yes | 47(45.6) | 10(34.5) |  |
| No | 56(54.4) | 19(65.5) |  |
| Interesting for brachytherapy |  |  | 0.613 |
| Without | 14(13.6) | 6(20.7) |  |
| With | 89(86.4) | 23(79.3) |  |
| Attending academic activities (times) |  |  | 0.903 |
| ≤2 | 77(74.8) | 23(79.3) |  |
| >2 | 26(25.2) | 6(20.7) |  |
| Taking brachytherapy very serious |  |  | 0.023 |
| No | 30(29.1) | 15(51.7) |  |
| Yes | 73(70.9) | 14(48.3) |  |

#: strong confidence to complete GBT was classified as the ability to perform GBT with minor assistance or complete it entirely independently

Table 2. Univariate and multivariate logistic regression analysis for self-reported competence of senior residents in finishing intracavity brachytherapy independently

| Variables | Univariate | | Multivariate | |
| --- | --- | --- | --- | --- |
|  | OR (95%CI for OR) | *p* | OR (95%CI for OR) | *p* |
| Workload of brachytherapy in SR-training unit(cases/year) | 1.000(0.999-1.001) | 0.855 |  |  |
| Performing intracavity brachytherapy cases (3D) |  |  |  |  |
| ≤10 | ref |  |  |  |
| >10 | ～ | 0.998 |  |  |
| Observating intracavity brachytherapy cases (3D) |  |  |  |  |
| ≤10 | ref |  | ref |  |
| >10 | 10.068(2.273-44.602) | **0.002** | 9.751(2.181-43.604) | 0.003 |
| Reading papers about brachytherapy |  |  |  |  |
| ≤10 | ref |  |  |  |
| >10 | 1.308(0.553-3.094) | 0.541 |  |  |
| Take caring of cases |  |  |  |  |
| ≤10 | ref |  |  |  |
| >10 | 0.767(0.262-2.242) | 0.628 |  |  |
| Reading MRI image cases |  |  |  |  |
| ≤10 | ref |  |  |  |
| >10 | 0.664(0.208-2.124) | 0.490 |  |  |
| Attending brachytherapy curriculum |  |  |  |  |
| No | ref |  |  |  |
| Yes | 1.294(0.475-3.527) | 0.614 |  |  |
| Interesting for brachytherapy |  |  |  |  |
| Without | ref |  |  |  |
| With | 1.640(0.568-4.737) | 0.361 |  |  |
| Attending academic activities (times) |  |  |  |  |
| ≤2 | ref |  |  |  |
| >2 | 1.294(0.475-3.527) | 0.614 |  |  |
| Taking brachytherapy very serious |  |  |  |  |
| No | ref |  | ref |  |
| Yes | 2.607(1.122-6.059) | **0.026** | 2.473(1.016-6.022) | 0.046 |

Abbreviations: Odd Ratios (OR) and 95% confidence intervals (CI) were calculated by a stratified logistic proportional hazards model.

Table 3. The factors related with self-reported competence of senior residents in finishing vaginal brachytherapy independently [M (range)/ n (%)]

| Variables | Group with strong confidence^#^ (N=99) | Group without confidence(N=33) | *p* |
| --- | --- | --- | --- |
| Workload of brachytherapy in SR-training unit(cases/year) | 600(180-1200) | 720(180-1050) | 0.852 |
| Performing vaginal brachytherapy cases (3D) |  |  | <0.001 |
| ≤5 | 68(68.7) | 33(100.0) |  |
| >5 | 31(31.3) | 0(0) |  |
| Observating vaginal brachytherapy cases (3D) |  |  | <0.001 |
| ≤5 | 50(50.5) | 29(87.9) |  |
| >5 | 49(49.5) | 4(12.1) |  |
| Take caring of cases |  |  | 0.901 |
| ≤10 | 20(20.2) | 7(21.2) |  |
| >10 | 79(79.8) | 26(78.8) |  |
| Reading MRI image cases |  |  | 1.000 |
| ≤10 | 18(18.2) | 6(18.2) |  |
| >10 | 81(81.8) | 27(81.8) |  |
| Reading papers about brachytherapy |  |  | 0.681 |
| ≤10 | 59(59.6) | 21(63.6) |  |
| >10 | 40(40.4) | 12(36.4) |  |
| Attending brachytherapy curriculum |  |  | 0.612 |
| No | 44(44.4) | 13(39.4) |  |
| Yes | 55(55.6) | 20(60.6) |  |
| Attending academic activities (times) |  |  | 1.000 |
| ≤2 | 75(75.8) | 25(75.8) |  |
| >2 | 24(24.2) | 8(24.2) |  |
| Taking brachytherapy very serious |  |  | 0.044 |
| No | 29(29.3) | 16(48.5) |  |
| Yes | 70 (70.7) | 17(51.5) |  |

#: strong confidence to complete GBT was classified as the ability to perform GBT with minor assistance or complete it entirely independently

Table 4.

Univariate and multivariate logistic regression analysis for self-reported competence of senior residents in finishing vaginal brachytherapy independently

| Variables | Univariate | | Multivariate | |
| --- | --- | --- | --- | --- |
|  | OR (95%CI for OR) | *p* | OR (95%CI for OR) | *p* |
| Workload of brachytherapy in SR-training unit(cases/year) | 1.000(0.999-1.001) | 0.890 |  |  |
| Performing vaginal brachytherapy cases (3D) |  |  |  |  |
| ≤5 | ref |  |  |  |
| >5 | ～ | 0.998 |  |  |
| Observating vaginal brachytherapy cases (3D) |  |  |  |  |
| ≤5 | ref |  | ref |  |
| >5 | 7.105(2.325-21.714) | **0.001** | 7.105(2.325-21.714) | 0.001 |
| Reading papers about brachytherapy |  |  |  |  |
| ≤10 | ref |  |  |  |
| >10 | 1.186(0.525-2.680) | 0.681 |  |  |
| Take caring of cases |  |  |  |  |
| ≤10 | ref |  |  |  |
| >10 | 1.063(0.404-2.800) | 0.901 |  |  |
| Reading MRI image cases |  |  |  |  |
| ≤10 | ref |  |  |  |
| >10 | 1.000(0.360-2.777) | 1.000 |  |  |
| Attending brachytherapy curriculum |  |  |  |  |
| No | ref |  |  |  |
| Yes | 1.692(0.845-3.391) | 0.138 |  |  |
| Attending academic activities (times) |  |  |  |  |
| ≤2 | ref |  |  |  |
| >2 | 1.000(0.399-2.508) | 1.000 |  |  |
| Taking brachytherapy very serious |  |  |  |  |
| No | ref |  |  |  |
| Yes | 2.272(1.012-5.098) | **0.047** |  |  |

Abbreviations: Odd Ratio (OR) and 95% confidence intervals (CI) were calculated by a stratified logistic proportional hazards model.

Table 5.

The relationship between the senior resident's competence in finishing GBT and the case numbers associated with GBT practice

| number of BT cases performed by residents | The percentage of senior residents having competence to perform brachytherapy independently or with minor assistance | | | | | | | | |
| --- | --- | --- | --- | --- | --- | --- | --- | --- | --- |
|  | Intracavity -2D/3D | Intracavity -2D | Intracavity -3D | Vaginal stump for EC -2D/3D | Vaginal stump for EC -2D | Vaginal stump for EC -3D | Interstitial 2D/3D | Interstitial 2D | Interstitial 3D |
| ＞50 | 100%(22/22) | 100(10/10) | 100%(12/12) | 100%(15/15) | 100%(5/5) | 100% (10/10) | 100%(5/5) | 100%(2/2) | 100%(3/3) |
| ＞40 | 100%(27/27) | 100%(13/13) | 100%(14/14) | 100%(19/19) | 100% (7/7) | 100% (12/12) | 100%(5/5) | 100%(2/2) | 100%(3/3) |
| ＞30 | 100%(38/38) | 100%(15/15) | 100%(18/18) | 100%(23/23) | 100% (9/9) | 100% (14/14) | 100%(13/13) | 100%(6/6) | 100%(7/7) |
| ＞20 | 100%(51/51) | 100%(20/20) | 100%(20/20) | 100%(37/37) | 100% (16/16) | 100% (21/21) | 100%(20/20) | 100%(9/9) | 100%(11/11) |
| ＞10 | 100%(53/53) | 100%(26/26) | 100%(27/27) | 100%(49/49) | 100% (21/21) | 100% (28/28) | **94.12%(32/33)** | **93.33%(14/15)** | **100%(18/18)** |
| ＞5 | **96%(74/70)** | **100%(35/35)** | **92.86%(39/42)** | 100%(65/65) | 100% (31/31) | 100% (34/34) | 85.11%(40/46) | 89.47%(17/19) | 85.19%(23/27) |
| ≥1 | 85.51%(118/138) | 88.73%(63/71) | 82.09%(55/67) | **84.96%(113/133)** | **88.24% (60/68)** | **81.54% (53/65)** | 64.49%(69/106) | 63.27%(31/49) | 66.67%(38/57) |
| 0 | 69.84%(88/126) | 65.57%(40/61) | 73.85%(48/65) | 64.89%(85/131) | 60.94% (39/64) | 68.66% (46/67) | 45.22%(71/158) | 46.99%(39/83) | 42.67%(32/75) |
| all | 78.03%(206/264) | 78.03%(103/132) | 78.03%(103/132) | 75%(198/264) | 75%(99/132) | 75%(99/132) | 53.03%(140/264) | 50.03%(70/132) | 50.03%(70/132) |

Table 6. The percentage of residents having confident in managing patients with following tumors independently.

|  | Confidence in managing patients according to guidelines, % (n/N) |
| --- | --- |
| Gynecologic tumor | 68.94 ( 91/132 ) |
| Breast cancer | 65.15 (86/132) |
| Tumor in lower digestive tract | 62.12 (82/132) |
| Thoracic Tumors | 56.06 (74/132) |
| Head and neck tumor | 38.64 (51/132) |
| Tumor in upper digestive tract | 37.88 (50/132) |
| lymphoma | 24.24 (32/132) |

**supplementary data: Appendix-1**

**A survey on brachytherapy training of gynecological cancer focusing on the self-reported competence of residents specialized radiation oncology.**

Mohan Dong^#^, Ph.D; Changhao Liu^#^, MD; Junfang Yan, MD; Yong Zhu, MD; Yutian Yin, MD; Jia Wang, MD; Lichun Wei, MD; Ying Zhang^🖂^, MD; Lina Zhao^🖂^, MD

Dear residents and physicians

The training necessary to become a doctor who provides radiotherapy for tumors is extremely valuable. In the future, your work will utilize brachytherapy and associated knowledge. The aim of this survey is to gain insight into the requirements for brachytherapy training and the potential methods for enhancing it. Currently, brachytherapy remains the most widely utilized treatment option for gynecological tumors. Hence, the scope of this research is restricted to the observation of training pertaining to the brachytherapy of gynecological tumors.

The evaluation of certain question is categorized into grades by using a 5-Likert-type scale, For example, very important, important, average, unimportant, unimportant.

The number of patients was actually observed, operated on, and managed: 0; 1-5； 6-10； 11-20； 21-30； 31-40； 41-50；＞51.

The number of literature studies is categorized as zero, one to ten, eleven to twenty, twenty-one to thirty, and thirty-one or more.

The self-reported competence was classified as follows: unable to perform BT, competence to perform BT with major assistance, indeterminacy, competence to perform BT with minor assistance, and competence to perform BT whole independently.

By engaging in thoughtful consideration and answering the questions provided in the questionnaire, it may exert a profound influence on your training. Please take a few minutes to make an accurate choice and return your choice to the department of radiation oncology of Xijing hospital via email within one week.

**Demographic information**

You are postgraduate year (PGY) resident, or junior staff employed for year

Gender: ；age : years old

specialized subject  ： radiation oncology； gynecologic oncology； oncology； radiology；

Location of standardized training unit ： province ; city

Location of work unit ： province ; city

Department engaged ： radiation oncology without subspecialty； gynecologic oncology of radiation oncology； radiation oncology of gynecologic oncology

Workload of gynecological tumor brachytherapy (GBT) in the training base where you are interning: cases / year

**questions related to brachytherapy**

1. Do you think it is important to be able to independently complete brachytherapy treatment (applicator insertion, target area delineation, primary review of treatment plan) at the end of resident training or rotation training in the radiotherapy department

Very important; Important; unclear; unimportance; Very unimportant

2. The leader or department director of the residential training program plays an important role in training your ability to carry out independent brachytherapy treatment

Very important; Important; unclear; unimportance; Very unimportant

3, At the end of your regular training, how confident are you in undergoing brachytherapy in following diseases?

The self-reported competence was classified as follows: unable to perform BT, competence to perform BT with major assistance, indeterminacy, competence to perform BT with minor assistance, and competence to perform BT whole independently.

| 2D, or 3D (both possible) | to perform BT whole independently | To perform BT with minor assistance | indeterminacy, | to perform BT with major assistance | unable to perform BT |
| --- | --- | --- | --- | --- | --- |
| Intracavitary bracherapy in radical radiotherapy for cervical cancer |  |  |  |  |  |
| Combined Intracavity and Interstitial brachytherapy in Radical Radiotherapy for Cervical Cancer |  |  |  |  |  |
| Postoperative Intravaginal cylinder for endometrial carcinoma |  |  |  |  |  |

4-1, During your training, the number of cases of the following diseases operated by yourself ( applicator insertion)

|  | 0 | 1-5 | 6-10 | 11-20 | 21-30 | 31-40 | 41-50 | ＞51 |
| --- | --- | --- | --- | --- | --- | --- | --- | --- |
| intracavitary brachytherapy for cervix (2D) |  |  |  |  |  |  |  |  |
| intracavitary brachytherapy for cervix ( image guided) |  |  |  |  |  |  |  |  |
| Combined Intracavity and Interstitial brachytherapy for cervix（2D） |  |  |  |  |  |  |  |  |
| Combined Intracavity and Interstitial brachytherapy for cervix（image guided） |  |  |  |  |  |  |  |  |
| Postoperative Intravaginal cylinder for endometrial carcinoma（2D） |  |  |  |  |  |  |  |  |
| Postoperative Intravaginal cylinder for endometrial carcinoma (image guided) |  |  |  |  |  |  |  |  |

4-2, During your training, observe the number of cases of the following brachytherapy ( applicator insertion)

|  | 0 | 1-5 | 6-10 | 11-20 | 21-30 | 31-40 | 41-50 | ＞51 |
| --- | --- | --- | --- | --- | --- | --- | --- | --- |
| intracavitary brachytherapy for cervix (2D) |  |  |  |  |  |  |  |  |
| intracavitary brachytherapy for cervix ( image guided) |  |  |  |  |  |  |  |  |
| Combined Intracavity and Interstitial brachytherapy for cervix（2D） |  |  |  |  |  |  |  |  |
| Combined Intracavity and Interstitial brachytherapy for cervix（image guided） |  |  |  |  |  |  |  |  |
| Postoperative Intravaginal cylinder for endometrial carcinoma（2D） |  |  |  |  |  |  |  |  |
| Postoperative Intravaginal cylinder for endometrial carcinoma (image guided) |  |  |  |  |  |  |  |  |

4-3 In the standardized training stage, the number of patients with gynecological tumors (cervical cancer, endometrial cancer, etc.) under your care

|  | 0 | 1-5 | 6-10 | 11-20 | 21-30 | 31-40 | 41-50 | ＞51 |
| --- | --- | --- | --- | --- | --- | --- | --- | --- |
| Manage patients（Historical data gathering、  physical examination and consultative care) |  |  |  |  |  |  |  |  |
| interpretation of MRI image |  |  |  |  |  |  |  |  |

5, With experience of more than 5 cases of intertissue implantation, we can independently perform intertissue implantation

Strongly agree; agree; unclear; disagree; Strongly disagree

If not, how many cases do you think it should be? Cases

6, With experience of more than 15 cases of intracavity brachytherapy, we can independently perform

strongly agree; agree; unclear; disagree; Strongly disagree

If not, how many cases do you think it should be? Cases

7, In the training of residents, the biggest obstacle hindering your ability to work independently with brachytherapy for gynecological tumors is

In the training process, the number of cases related to the department is insufficient

In the training process, the instructor did not give practical operation opportunities

Lack of appropriate teaching/process training

Individuals lack interest in brachy therapy

8, At the end of the regular training or at the end of the rotation of the radiotherapy department, your Competency to carry out brachytreatment independently passed the evaluation of the resident training teachers

Adoption; Fail to pass; No comments

9, In my residential training program, is there formal brachytherapy teaching course ?

Yes; No

10, Do you think brachytherapy should have its own formal training courses?

Very unnecessary, unnecessary, unclear, should have, very necessary

11-1, After you have completed the residential training program, how confident are you in finishing following works?

|  | to finish whole independently | To finish with minor assistance | indeterminacy, | To finish with major assistance | unable to finish |
| --- | --- | --- | --- | --- | --- |
| Brachytherapy for gynecologic cancer  ( 2D or image guided） |  |  |  |  |  |
| SBRT/SRS (tumor with any location) |  |  |  |  |  |
| interpretation of MRI image |  |  |  |  |  |
| Historical data gathering、physical examination and consultative care |  |  |  |  |  |

11-2 When you complete the residential training project, how confident are you in finishing the treatment of following disease? (including patient care, target delineation, plan evaluation).

|  | to finish whole independently | To finish with minor assistance | indeterminacy, | To finish with major assistance | unable to finish |
| --- | --- | --- | --- | --- | --- |
| Head and neck tumor(such as Nasopharyngeal cancer, oropharyngeal cancer) |  |  |  |  |  |
| Thoracic tumors (such as lung cancer and esophageal cancer) |  |  |  |  |  |
| Upper digestive tract tumors (such as pancreatic cancer and liver cancer) |  |  |  |  |  |
| Tumors of lower digestive tract (such as rectal cancer, anal cancer) |  |  |  |  |  |
| Gynecological tumors (such as cervical cancer and endometrial cancer) |  |  |  |  |  |
| lymphoma |  |  |  |  |  |
| Breast cancer |  |  |  |  |  |

11-3 How many articles related to brachytherapy have you read during your resident training or rotation in the radiotherapy department

0； 1-10； 11-20； 21-30； ＞30

12-1, you have participated in the work for year, and now you have the opportunity to independently complete the following tasks. Your confidence in completing this task is ( for junior doctors participating in gynecological tumor radiotherapy)

|  | Very low | low | neutral | high | Very high |
| --- | --- | --- | --- | --- | --- |
| Brachytherapy for gynecologic cancer  （2D or image guided） |  |  |  |  |  |
| interpretation of MRI image about gynecologic cancer |  |  |  |  |  |
| Historical data gathering、physical examination and consultative care |  |  |  |  |  |

12-2 You have participated in the work for year, and have completed the following cases independently or under the guidance of the instructor ( for junior doctors participating in gynecological tumor radiotherapy)

|  | 0 | 1-5 | 6-10 | 11-20 | 21-30 | 31-40 | 41-50 | ＞51 |
| --- | --- | --- | --- | --- | --- | --- | --- | --- |
| intracavitary brachytherapy for cervix (2D) |  |  |  |  |  |  |  |  |
| intracavitary brachytherapy for cervix ( image guided) |  |  |  |  |  |  |  |  |
| Combined Intracavity and Interstitial brachytherapy for cervix（2D） |  |  |  |  |  |  |  |  |
| Combined Intracavity and Interstitial brachytherapy for cervix（image guided） |  |  |  |  |  |  |  |  |
| Postoperative Intravaginal cylinder for endometrial carcinoma（2D） |  |  |  |  |  |  |  |  |
| Postoperative Intravaginal cylinder for endometrial carcinoma (image guided) |  |  |  |  |  |  |  |  |

12-3 You have been working for years, and how many articles related to brachytherapy have you read y during your work?

0； 1-10； 11-20； 21-30； ＞30

13, Please evaluate the role of brachytherapy in the following diseases in the next 10 years

|  | Likely to decline | remain unchanged | Likely to increase |
| --- | --- | --- | --- |
| Radical radiotherapy for cervical cancer |  |  |  |
| Postoperative radiotherapy for endometrial carcinoma |  |  |  |

14, The use of brachytherapy has declined in many diseases. Do you think the problem is ？

Not serious at all; not serious; not clear; some serious; very serious

15, What aspects should the national cancer radiotherapy professional committee support brachytherapy training and education? (Multiple choices are allowed) ？

Technical training

More brachytherapy education projects at the annual meeting

Design and promote comprehensive education curriculum related to brachytherapy

Online teaching mode

Other

16, Which of the following questions do you think is the most important in the education training of brachytherapy training ？

brachytherapy education projects at the annual academic conference

comprehensive education curriculum related to brachytherapy

Online teaching mode

Other

17， During your residency training, which activities related with brachytherapy did you participate in? (Multiple choices are allowed)

International conference related brachytherapy content

National academic annual meeting on brachytherapy

Teaching courses organized by national associations (comprehensive brachytherapy, gynecology, prostate, physics)

Provincial training program

18. In the process of resident training, if you do not have the ability to work independently in brachytherapy, what activities will you participate in in the future to rapidly improve your ability to perform brachytherapy (single choice)

Large number of practical operation opportunities

Have good teacher

Formal training courses and technical training (theory, phantom simulation)

International conference: relevant content of brachytherapy

National academic annual meeting: content of brachytherapy

Provincial training program

19 In order to improve the accuracy and quality control of applicator insertion and workflow , is simulation-based graduate medical education necessary?

Very unnecessary, unnecessary, unclear, necessary, very necessary

20，Do you have any suggestions or comments on how to improve brachytherapy training?

**supplementary data: Appendix-2**

**A survey on brachytherapy training of gynecological cancer focusing on the self-reported competence of residents specialized radiation oncology.**

Mohan Dong^#^, Ph.D; Changhao Liu^#^, MD; Junfang Yan, MD; Yong Zhu, MD; Yutian Yin, MD; Jia Wang, MD; Lichun Wei, MD; Ying Zhang^🖂^, MD; Lina Zhao^🖂^, MD

Supplementary table: The list of the leader and manager of training units responsible for resident GBT training

|  | Teachers | Units for GBT training |
| --- | --- | --- |
|  | Ji Ouyang  Xinping Cao | Department of Radiation Oncology, Sun Yat-sen University Cancer Center, State Key Laboratory of Oncology in South China |
|  | Rao Xing | Department of Radiation Oncology, Yunnan Cancer Hospital, the Third Affiliated Hospital of Kunming Medical University |
|  | Hong Zhao | Department of Radiation Oncology, the Affiliated Hospital of Yan'an University |
|  | Junfang Yan  Fuquan Zhang | Department of Radiation Oncology, the Peking Union Medical College Hospital, Chinese Academy of Medical Science and Peking Union Medical College |
|  | Xiaoting Xu | Department of Radiation Oncology, the First Affiliated Hospital of Soochow University |
|  | Yunfeng Mu  Qiufang Liu | Department of Radiation Oncology, Shaanxi Provincial Cancer Hospital |
|  | Shuhong Zhao  Qing Wang | Department of Radiation Oncology, Shaanxi Provincial People's Hospital |
|  | Jianli He | Department of Radiation Oncology, General Hospital of Ningxia Medical University |
|  | Xiaoge Sun | Department of Radiation Oncology, Affiliated Hospital of Inner Mongolia Medical University |
|  | Qinglian Wen | Department of Radiation Oncology, Affiliated Hospital of Southwest Medical University |
|  | Jin Su  Zi Liu | Department of Radiation Oncology,the First Affiliated Hospital of Xi'an Jiaotong University |
|  | Guanghui Cheng | Department of Radiation Oncology, China-Japan Union Hospital of Jilin University |
|  | Jianjun Zhang  Rutie Yin | Department of Gynecologic Oncology, West China Second University Hospital, Sichuan University, Chengdu, China/Key Laboratory of Obstetrics & Gynecologic and Pediatric Diseases and Birth Defects of Ministry of Education, West China Second University Hospital, Sichuan University |
|  | Yunyan Zhang | Department of Radiation Oncology, Affiliated Tumor Hospital of Harbin Medical University |
|  | Mingchun Zhang  Shuxia Cheng | Department of Gynecologic Oncology, Affiliated Cancer Hospital of Zhengzhou University |
|  | Xiangkun Yuan | Department of Radiation Oncology, Cangzhou Hospital of Integrated Traditional Chinese and Western Medicine |
|  | Kuixiu Li | Gynecology and Oncology Department, Fourth Hospital of Hebei Medical University |
|  | Hanmei Lou | Gynecology and Oncology Department, the Cancer Hospital of the University of Chinese Academy of Sciences (Zhejiang Cancer Hospital), Institute of Basic Medicine and Cancer (IBMC), Chinese Academy of Sciences |
|  | Yijing Ye | Department of Radiation Oncology, Zhongshan People's Hospital |
|  | Fengju Zhao | Department of Radiation Oncology, Gansu Provincial Cancer Hospital |
|  | Wenjun Chen | Department of Radiation Oncology, Fujian Provincial Cancer Hospital |
|  | Lijuan Zou | Department of Radiation Oncology, the Second Affiliated Hospital of Dalian Medical University |
|  | Ping Jiang | Department of Radiation Oncology, the Third Affiliated Hospital of Peking University |
|  | Xiaofan Li | Department of Radiation Oncology, the Affiliated Cancer Hospital of Peking University |
|  | Yong Zhu | Department of Radiation Oncology, Baoji Central Hospital |
|  | Shaojun Chen | Department of Radiation Oncology, the Fourth Affiliated Hospital of Guangxi Medical University |
|  | Lichun Wei  Lina Zhao | Department of radiation oncology, Xijing Hospital, Air Force Medical University |
|  | Gulina Kuerban | Department of radiation oncology, Affiliated Cancer Hospital of Xinjiang Medical University |
